# Synthesis and Evaluation of High-Temperature-Resistant and Environmentally Friendly Polymer Filter Loss Additives

**DOI:** 10.3390/polym17060792

**Published:** 2025-03-17

**Authors:** Ming Tian, Chuan Yang, Qian Huang, Ruixue Wang, Xiaoming Su, Peng Xu, Tao Peng

**Affiliations:** 1 CNPC Tarim Oilfield Branch, Korla 841000, China; tianming741@126.com (M.T.); yangchuan963@126.com (C.Y.); huang525x@163.com (Q.H.); wrx250dl@126.com (R.W.); suxiaoming2042@163.com (X.S.); 2 Xinjiang Key Laboratory of Ultra-Deep Oil and Gas, Korla 841000, China; 3 R&D Center for Ultra Deep Complex Reservior Exploration and Development, Tarim Oilfield Branch of CNPC, Korla 841000, China; 4 School of Peili Petroleum Engineering, Lanzhou City College, Lanzhou 730070, China; 5 School of Petroleum Engineering, Yangtze University, Wuhan 430102, China

**Keywords:** fluid loss additive, environmental friendliness, high-temperature resistance, polymer, water-based drilling fluid

## Abstract

In the process of oil extraction, the drilling fluid, as a critical operational fluid, directly impacts the drilling efficiency and safety. However, under high-temperature and high-pressure conditions, the drilling fluid tends to experience fluid loss, which not only causes environmental pollution but also increases the drilling costs and challenges. To address this issue, this study aimed to develop a novel high-temperature-resistant and environmentally friendly polymer fluid loss additive—EnSipoly-FL—designed to enhance the thermal stability and environmental performance of drilling fluids. The copolymerization of acrylamide (AM), N-vinylpyrrolidone (NVP), acrylic acid (AA), and vinyltrimethoxysilane (A-171) was selected to ensure the thermal and chemical stability of the fluid loss additive. The synthesis conditions, including the initiators, emulsifiers, reaction temperature, and time, were optimized in the experiments. The structure of the target product was confirmed by infrared spectroscopy and nuclear magnetic resonance analysis. Thermogravimetric analysis and particle size analysis demonstrated that the polymer possessed excellent thermal stability and appropriate physical dimensions. Environmental impact assessments indicated that EnSipoly-FL exhibited good biodegradability and low toxicity, meeting environmental protection standards. Comprehensive performance tests showed that the polymer microspheres exhibited exceptional fluid loss reduction capabilities and environmental friendliness in high-temperature and high-pressure drilling fluid applications. This makes it a promising candidate for widespread use in the oil drilling industry, advancing the green development of drilling fluid technology.

## 1. Introduction

With the continuous advancement of oil exploration and extraction technologies, drilling operations in high-temperature and high-pressure environments have become more prevalent. As a result, the performance requirements for drilling fluid additives, particularly fluid loss control agents, have become increasingly stringent. These additives must not only withstand extreme thermal conditions but also maintain their effectiveness in reducing fluid loss, ensuring wellbore stability, and enhancing the overall drilling efficiency [1,2,3,4]. Traditional fluid loss additives tend to degrade under high-temperature conditions, resulting in decreased drilling fluid performance, failing to meet the demands of high-temperature drilling [5,6]. Meanwhile, the growing emphasis on environmental protection necessitates reductions in environmental pollution during the production and application of fluid loss additives [7,8]. Consequently, the development of advanced, high-performance fluid loss additives that exhibit superior thermal stability and environmental compatibility has become a critical focus in modern drilling fluid research [9,10,11]. Currently, the fluid loss additives on the market can be broadly categorized into two types: natural polymers and synthetic polymers. Natural polymers, such as carboxymethyl cellulose (CMC) and plant-derived gums, are widely used due to their low costs, abundant availability, and environmental friendliness [12,13]. However, these natural polymers are prone to thermal degradation under high-temperature conditions, leading to a significant decline in their rheological properties and fluid loss control performance, making them less suitable for demanding high-temperature drilling operations [14,15]. Synthetic polymers, such as polyacrylamide (PAM), exhibit better high-temperature resistance, but their high synthesis costs, poor biodegradability, and environmental pollution issues remain significant challenges [16,17,18].

In terms of monomer selection and synthesis methods, researchers in recent years have conducted extensive studies on the development of high-temperature-resistant and environmentally friendly polymer fluid loss additives [19,20,21,22]. For monomer selection, researchers tend to choose monomers with excellent thermal stability and environmental characteristics, such as acrylamide, 2-acrylamido-2-methylpropanesulfonic acid (AMPS), and itaconic acid, among others [23,24,25]. These monomers not only exhibit good thermal stability but also help to reduce the overall cost of the polymer to a certain extent. In terms of synthesis methods, researchers have developed and applied various advanced polymerization techniques to optimize the performance of fluid loss additives [26,27,28]. Currently, common synthesis methods include emulsion polymerization, free radical polymerization, and ionic polymerization [29,30,31]. By optimizing the polymerization conditions, including the temperature, time, and pressure, researchers have successfully synthesized polymer fluid loss additives with superior performance. The structure and properties of polymers are key factors influencing their fluid loss performance. Researchers have adjusted polymers’ structures and properties by modifying parameters such as the molecular weight, the molecular weight distribution, and the type and quantity of functional groups [32,33,34]. For example, incorporating thermally stable functional groups, such as sulfonic acid or carboxylic acid groups, can enhance the thermal stability of the polymer [35,36]. Similarly, adjusting the molecular weight distribution of the polymer can improve its solubility and dispersibility, thereby enhancing its fluid loss reduction performance [37,38].

High-temperature resistance is one of the most critical properties of high-temperature-resistant and environmentally friendly polymer fluid loss additives. Researchers evaluate this property by testing indicators such as the fluid loss performance and viscosity changes under high-temperature conditions [5,39,40]. Additionally, long-term stability tests simulating high-temperature drilling conditions are conducted to assess the additives’ thermal stability in practical applications. Environmental performance is another essential characteristic of these polymer fluid loss additives [41,42]. Researchers assess the environmental performance of polymers by analyzing key indicators, including pollutant emissions generated during production and application, as well as the biodegradability and eco-friendliness of the materials. These evaluations help to ensure that polymer-based additives meet sustainability standards while maintaining their effectiveness in various industrial applications [43,44,45]. Efforts are also being made to explore the use of renewable resources and environmentally friendly raw materials in the production of polymer fluid loss additives to minimize their environmental impact [46,47]. Traditional fluid loss additives, such as carboxymethyl cellulose (CMC)-based products, perform well under normal temperature conditions but degrade easily under high temperatures, leading to performance deterioration [43,48,49]. Furthermore, these conventional products have limitations in terms of environmental protection, as they may contain components that are harmful to the environment [50,51]. Therefore, developing a new fluid loss additive that combines high-temperature resistance with excellent environmental performance is of great significance for the sustainable development of the oil industry.

This study aimed to synthesize a high-temperature-resistant and environmentally friendly polymer fluid loss additive and evaluate its performance. Polymers with good thermal stability and biodegradability were selected as base materials, and, through molecular design and synthesis strategies, a series of novel fluid loss additives was prepared. These additives demonstrated outstanding stability and fluid loss performance under high-temperature conditions, while also exhibiting excellent environmental characteristics in biodegradability tests.

## 2. Materials and Methods

### 2.1. Experimental Materials

Acrylamide (AM), acrylic acid (AA), 1-vinyl-2-pyrrolidone (NVP), vinyltrimethoxysilane (A-171), ammonium persulfate (APS, analytical grade), sodium bisulfite (NaHSO_3_, analytical grade), and sodium hydroxide (NaOH) were purchased from Shanghai Macklin Biochemical Co., Ltd. (China). Emulsifiers (SF2810, Tween80, Span80) and heptane were procured from Sinopharm Chemical Reagent Co., Ltd. Deionized water was prepared in the laboratory, and sodium bentonite was obtained from Jingzhou Jiahua Technology Co., Ltd. (China).

### 2.2. Experimental Setup

Constant-temperature magnetic stirrer (NYP19-2), manufactured by Shanghai Meiyingpu Instrument Co., Ltd., China; six-speed rotational viscometer and high-temperature high-pressure filtration loss tester, manufactured by Qingdao Haitongda Instrument Co., Ltd., China; electronic constant-temperature stainless-steel water bath, manufactured by Shanghai Yulong Instrument Equipment Co., Ltd., China; optical microscope, manufactured by Nanjing Jiangnan Yongxin Optics Co., Ltd., China; scanning electron microscope, manufactured by Hitachi, Japan; laser particle size analyzer, manufactured by Zhuhai OMEC Instruments Co., Ltd., China; thermogravimetric analyzer, manufactured by Guangdong Gester Instruments Technology Co., Ltd., China.

### 2.3. Inverse Micro-Lotion Polymerization Method

In a four-neck flask equipped with a thermometer, stirrer, and reflux condenser, a certain amount of liquid heptane and emulsifier was added. Nitrogen gas was introduced for protection, and the mixture was stirred at a constant rate to achieve thorough emulsification, serving as the oil-phase solution. In a beaker, a certain amount of acrylic acid (AA) was first dissolved in distilled water, and the pH of the system was adjusted using NaOH to obtain a sodium salt aqueous solution of AA. Then, according to the formulation, acrylamide (AM), N-vinylpyrrolidone (NVP), and a coupling agent (A-171) were added at a certain mass ratio and stirred thoroughly until fully dissolved to obtain the aqueous-phase solution. The prepared aqueous-phase solution was then slowly added to the oil-phase solution. At room temperature, the mixture was emulsified using a high-shear mixer at high speed for 30 min to obtain a stable reverse-phase microemulsion. The resulting reverse-phase microemulsion was placed in a constant-temperature water bath under nitrogen protection, and the temperature was slowly raised to the specified level. An initiator was added dropwise to initiate the polymerization reaction. After a certain period, when the reaction was complete, it was stopped to obtain the microemulsion polymerization fluid loss reducer product, EnSipoly-FL. A portion of the obtained microemulsion product was demulsified and precipitated with ethanol, followed by multiple ethanol washings to remove surfactants. The solid polymer product was then vacuum-dried at 60 °C for 24 h, ground into a powder, and stored in a desiccator for later use.

### 2.4. Principles and Advantages of Synthesis

Polymer microspheres have the advantages of a controllable particle size distribution, simple preparation methods, low costs, and high strength, making them highly promising for applications in drilling fluids. Polymer microspheres can achieve fluid loss reduction through mechanisms such as adsorption, accumulation, and filling. Using reverse-phase microemulsion polymerization, which differs from the “water-in-oil” system of emulsion polymerization, reverse-phase emulsion polymerization involves dispersing an aqueous solution of monomers into an oil medium with the help of emulsifiers. The resulting product consists of polymer particles swollen with water. Reverse-phase emulsion polymerization offers a high polymerization rate, higher solid content compared to products from aqueous solution polymerization, fast heat dissipation, and good water solubility in the product. It also produces particles with a unique core–shell structure, where the center is a rigid crosslinked core and the outer layer is a shell containing hydrophilic groups. This spherical aggregated structure has high thermal stability and is minimally affected by the temperature. The rigid crosslinked core in the center resembles the main chain of hyperbranched polymers and can serve as a secondary structural carrier, enabling the spatial hierarchical distribution of hydrophilic groups.

Selecting suitable monomers is critical in preparing high-temperature-resistant and environmentally friendly polymer fluid loss additives. The monomers chosen in this study included acrylamide (AM), N-vinylpyrrolidone (NVP), acrylic acid (AA), and vinyltrimethoxysilane (A-171). These monomers were selected based on their specific properties, working synergistically to impart the desired comprehensive performance to the final product. Acrylamide (AM) provides excellent hydrophilicity and hydrolysis resistance. The hydrogen bonds formed between amide groups and water molecules enhance the stability of the polymer. N-vinylpyrrolidone (NVP), due to its rigid five-membered lactam group, significantly improves the rigidity of the copolymer chain, while inhibiting the hydrolysis of amide groups, ensuring stable performance under high-temperature and saline-/calcium-rich conditions. Acrylic acid (AA), with its carboxylic acid groups, enhances the hydration and adsorption capacity, positively contributing to the fluid loss reduction performance and stability of the polymer. Vinyltrimethoxysilane (A-171) introduces organosilane groups, which hydrolyze in alkaline drilling fluids to form silanol groups. These silanol groups condense with silanol groups on the surfaces of clay particles, thereby enhancing the adsorption stability of the polymer on clay.

### 2.5. Basic Performance Evaluation Methods

Base Slurry Preparation: Measure 350 mL of distilled water and place it in a cup. Add 0.79 g (accurate to 0.01 g) of anhydrous sodium carbonate and 10.85 g (accurate to 0.01 g) of drilling fluid test bentonite (in accordance with SY 5490-2016 standards [52] for drilling fluid test clay). Stir the mixture at high speed for 20 min, pausing at least twice during this time to scrape down clay adhering to the walls of the container. Allow the mixture to age in a sealed container at room temperature for 24 h to form the base slurry.

Add a certain amount (accurate to 0.01 g) of the fluid loss additive sample to the base slurry. Stir the mixture at high speed for 20 min, pausing at least twice to scrape down the sample adhering to the walls of the container. Then, add 0.79 g (accurate to 0.01 g) of NaOH granules to adjust the pH. Transfer the drilling fluid into a high-temperature aging cell and subject it to rolling aging at 200 °C for 16 h. After aging, remove the high-temperature cell, allow it to cool, and then open it. Stir the mixture at high speed for 5 min. Measure the medium-pressure fluid loss (at room temperature) of the drilling fluid following the procedure outlined in Section 7.2 of GB/T 16783.1-2014 [53]. The specific steps are as follows. Pour the thermally rolled drilling fluid into the fluid loss tester’s drilling fluid cup (ensure that all components of the drilling fluid cup, especially the filter screen, are clean and dry and confirm that the sealing ring is neither deformed nor damaged). The fluid level should be 1–1.5 cm below the top of the drilling fluid cup. Place a dry graduated cylinder under the discharge pipe to collect the filtrate. Close the pressure release valve and adjust the pressure regulator to reach 690 kPa ± 35 kPa within 30 s or less, starting the timer simultaneously as pressure is applied. After 30 min of applying pressure, measure the volume of filtrate collected, denoted as FL (API) (i.e., medium-pressure fluid loss). The performance of the fluid loss additive is evaluated based on the FL (API) value.

## 3. Results and Discussion

### 3.1. Determination of Optimal Synthesis Conditions

The performance of spherical polymer fluid loss additives is closely related to their synthesis conditions. Since the AM/AA/NVP/A-171 quaternary copolymer is prepared through reverse-phase microemulsion polymerization, the main influencing factors include the oil-to-water ratio of the microemulsion, the pH value of the reaction system, the amount of emulsifier, the amount of initiator, the total monomer concentration, the ratio between monomers, the reaction temperature, and the reaction time.

#### 3.1.1. Different Oil-to-Water Ratios and the Stability of Microemulsions

The oil-to-water ratio has a significant impact on the stability of the reverse-phase microemulsion system. Therefore, with the emulsifier SF2810 (amine type) fixed at 1%, the effect of different oil-to-water ratios on the stability of the microemulsion system was investigated, as shown in Table 1. It can be observed that as the oil-to-water ratio increases, the stability of the system decreases. When the oil-to-water ratio is 5:4, a uniform, transparent, and stable microemulsion is obtained. Therefore, the optimal oil-to-water ratio for this microemulsion system is 5:4.

#### 3.1.2. Reaction System pH and the Performance of Fluid Loss Additives

**Figure 1:**
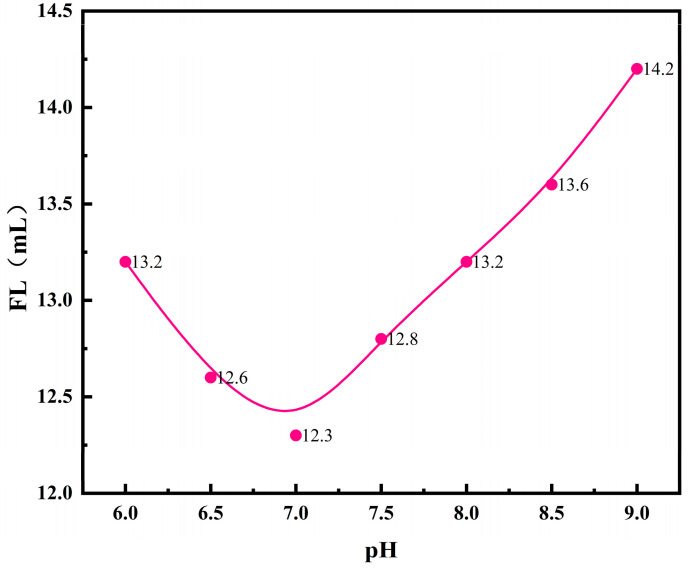
Effects of reaction system pH on the performance of fluid loss additives.

The initiator selected for the reaction system is a redox initiator composed of ammonium persulfate and sodium bisulfite, which exhibits acidity after ionizing in water. Additionally, the polymerization monomer contains acrylic acid, which ionizes hydrogen ions in water. Therefore, its decomposition rate is greatly influenced by the pH value of the system, which in turn affects the fluid loss performance of the copolymer product. To determine the optimal pH value for the reaction system, the pH was varied while keeping the other synthesis conditions constant. The main synthesis conditions were as follows: the initiator amount was 0.2% of the monomers, the monomer concentration was 25% (the ratio of solute to solution), and the molar ratio of monomers was AM:AA:NVP:A-171 = 9:4:3:2. The reaction temperature was 60 °C, and the reaction time was 5 h. The oil phase consisted of 56 mL of heptane, with 15 g of the SF2810 (amine-based) emulsifier. The system pH was set to 7, and the aqueous phase consisted of 45 mL of deionized water. Other conditions remained constant, except for those optimized later. The FL (API) of the base slurry system after adding the copolymer product is shown in Figure 1.

#### 3.1.3. Emulsifiers and the Performance of Fluid Loss Additives

**Figure 2:**
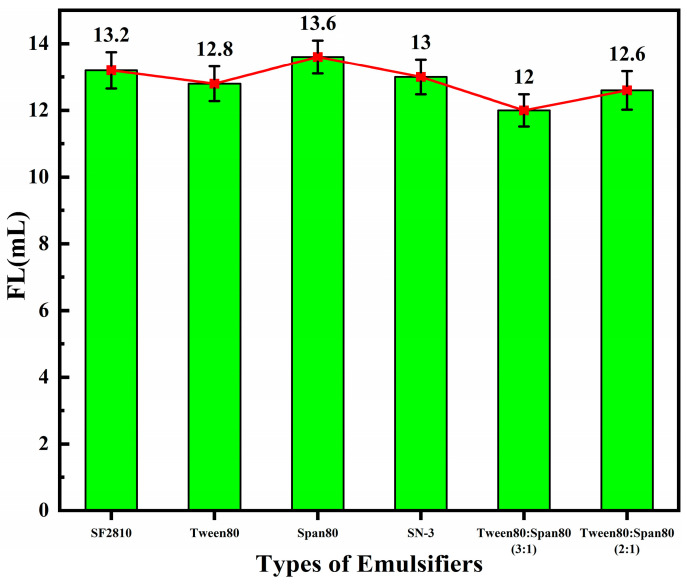
Effects of emulsifiers on the performance of fluid loss additives.

Microemulsions are thermodynamically stable systems stabilized by surfactants. The size and number of various particles are closely related to the amount of emulsifier used. Therefore, the type of emulsifier determines the stability of the system and affects the performance of the copolymer microspheres. By varying the type of emulsifier while keeping the other synthesis conditions constant, the FL (API) of the base slurry system after adding the copolymer product was obtained, as shown in Figure 2.

As shown in Figure 2, among the four emulsifiers used individually, Tween80 was the most effective as the emulsifier for the preparation of microsphere polymers via reverse-phase emulsion polymerization. The fluid loss additive prepared using Tween80 reduced the FL (API) of the base slurry system most significantly, with a fluid loss volume of 12.8 mL. The emulsifying and solubilizing capabilities of blended emulsifiers greatly exceed those of any single emulsifier. Not only can blended emulsifiers improve the emulsification efficiency, but they can also reduce the required amount of emulsifier. Additionally, the mutual penetration of blended emulsifiers can increase the flexibility of the interfacial layer, which facilitates the formation of microemulsions. The experiments showed that the fluid loss additive prepared using blended emulsifiers exhibited better fluid loss reduction performance. When the ratio of Tween80 to Span80 was 3:1, the FL (API) of the base slurry system decreased to 12 mL. Therefore, the non-ionic emulsifiers Tween80 and Span80, whose emulsifying effects are not affected by the pH, were selected as the composite emulsifier.

**Figure 3:**
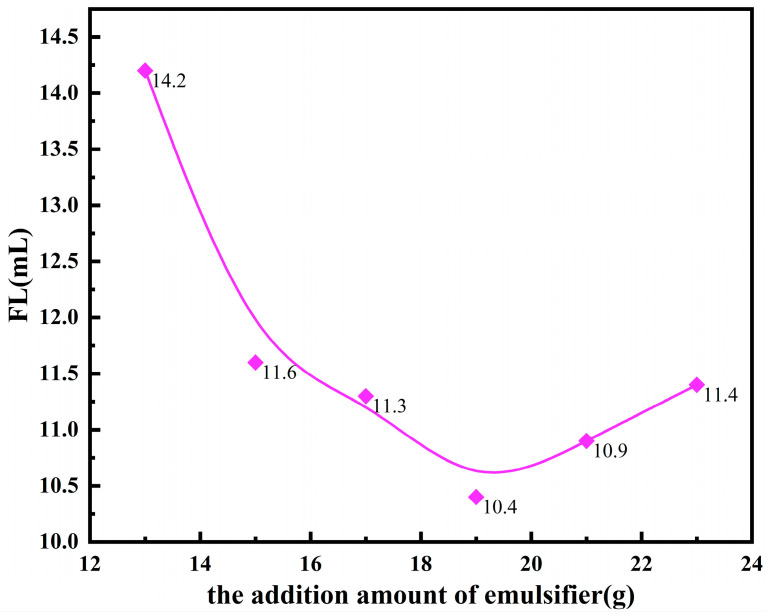
Effects of the emulsifier dosage on the performance of fluid loss additives.

During the reverse microemulsion polymerization process, the effect of the emulsifier on the properties of microsphere polymers was examined by varying the amount of emulsifier added, while keeping the other synthesis conditions constant. Only the amount of emulsifier (Tween80/Span80 = 3:1) was altered. The FL (API) of the base slurry system after the addition of the copolymer product is shown in Figure 3.

From Figure 3, it can be seen that as the amount of emulsifier (Tween80/Span80 = 3:1) increases, it facilitates the stabilization of the microemulsion (water-in-oil type) and promotes the preparation of microsphere polymer filtrate reducers. In the water-in-oil system, the rigidly crosslinked inner core formed by polymerized monomers features a unique core–shell structure, providing excellent temperature resistance. The outer shell, which is composed of hydrophilic groups, undergoes chemical adsorption with clay, enhancing the stability of the drilling fluid. When the amount of emulsifier added reaches 19 g, the FL (API) of the base slurry system decreases to 10.4 mL. However, when the emulsifier amount exceeds 19 g, the filtrate loss reduction effect diminishes. Therefore, the optimal emulsifier (Tween80/Span80 = 3:1) dosage for the synthesis of the copolymer filtrate reducer is determined to be 19 g.

#### 3.1.4. Initiator Dosage and the Performance of Fluid Loss Additives

**Figure 4:**
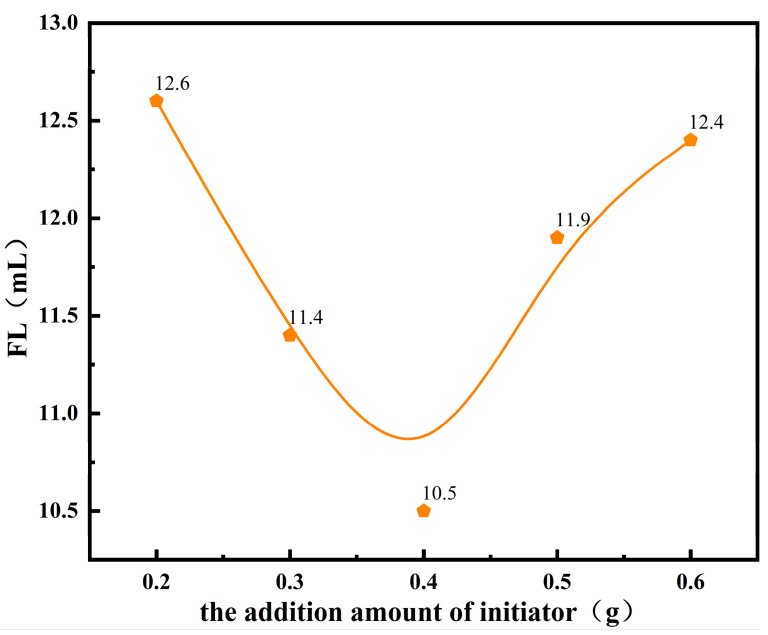
Effects of the initiator dosage on the performance of fluid loss additives.

The amount of initiator directly affects the average molecular weight and molecular structure of the copolymer, which in turn influences the filtrate loss reduction performance of the product. Through comparative experiments, while keeping the other synthesis conditions constant, the effect of the ammonium persulfate–sodium bisulfite initiator dosage (as a percentage of the total monomer mass) on the filtrate loss performance of the product was studied. The FL (API) of the base slurry system after the addition of the copolymer product is shown in Figure 4.

From Figure 4, it can be observed that, with an increase in the initiator dosage, the FL (API) of the drilling fluid system initially decreases and then increases. When the initiator dosage reaches 0.4% of the monomer mass, the FL (API) achieves its minimum value. This is because, when the initiator dosage is too low, the number of primary free radicals generated by its decomposition is insufficient. These radicals become surrounded by a large number of solvent molecules, forming a “cage effect”, which makes it difficult for the radicals to initiate monomer polymerization and makes them more prone to self-coupling termination. This results in low initiation efficiency, an insufficient copolymer concentration in the solution, and an inability to control filtrate loss. According to the free radical polymerization mechanism, the degree of polymerization of the product is inversely proportional to the square root of the initiator concentration. Thus, when the initiator concentration is too high, the average molecular weight of the copolymer becomes smaller, and the molecular structure contains excessive branching, which is also unfavorable for the control of water loss. Therefore, the optimal initiator dosage is determined to be 0.4% of the total monomer mass.

#### 3.1.5. Monomer Mass Fraction and the Performance of Fluid Loss Additives

**Figure 5:**
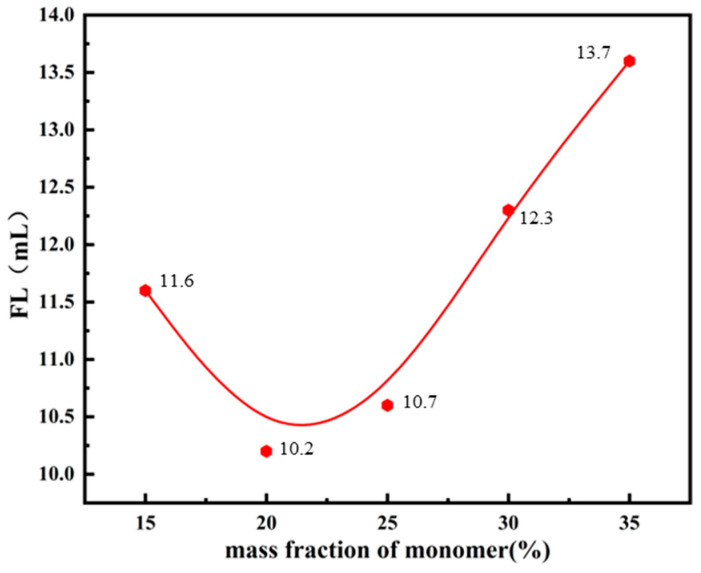
Effects of the monomer mass fraction on the performance of fluid loss additives.

According to the principles of free radical polymerization, the average degree of polymerization of the product is not only inversely proportional to the square root of the initiator concentration but also directly proportional to the square root of the total monomer concentration. Therefore, the characteristics of the product are significantly influenced by the total monomer concentration used in the reaction. To study the effect of the total monomer concentration on the filtrate loss reduction performance of microsphere polymers, the filtrate loss performance of the product was measured under different total monomer concentrations, while keeping the other reaction conditions constant. The FL (API) of the base slurry system after adding the copolymer product is shown in Figure 5.

From Figure 5, it can be observed that, as the total monomer concentration increases, the FL (API) of the base slurry system after adding the copolymer microsphere product initially decreases, reaching its lowest point when the total monomer concentration is 20%. However, as the monomer concentration continues to increase, the filtrate loss begins to rise significantly. This phenomenon can be explained by the fact that the average degree of polymerization of the product is directly proportional to the square root of the total monomer concentration. When the monomer concentration is relatively low, the average degree of polymerization of the product is limited, resulting in an insufficient kinetic chain length. Consequently, the product cannot effectively adsorb clay particles through encapsulation and bridging effects, which restricts its ability to stabilize the colloid. However, excessively increasing the total monomer concentration leads to difficulties in dissipating the reaction heat generated during the copolymerization process. This significantly enhances the auto-acceleration effect, causing the reaction system’s viscosity to become excessively high. As a result, the mobility of active chain ends is hindered, and they may even become trapped by surrounding molecules, preventing effective interaction and reaction. This increases the probability of chain termination, reduces the average degree of polymerization, and consequently limits the filtrate loss reduction performance of the copolymer. Based on optimization experiments at different concentrations, the optimal total monomer concentration is determined to be 20%.

#### 3.1.6. Monomer Molar Ratio and the Performance of Fluid Loss Additives

**Figure 6:**
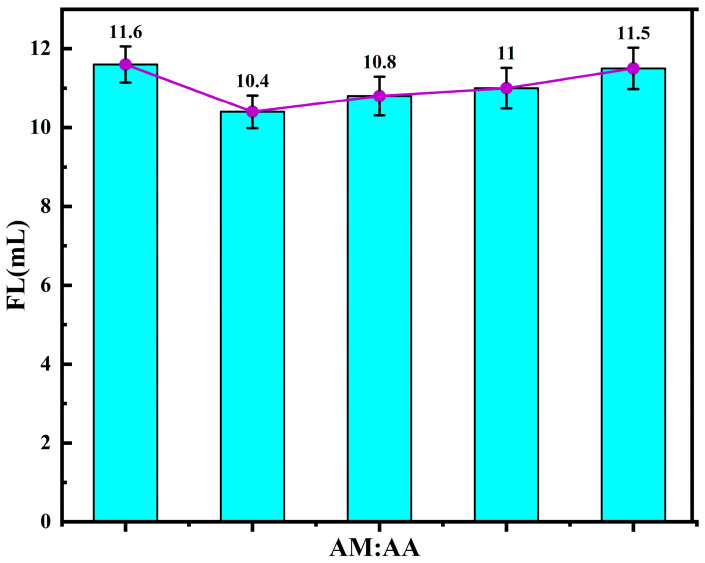
Effects of the AM-to-AA molar ratio on the performance of fluid loss additives.

During the synthesis of quaternary copolymers, the ratio of individual monomers has a significant impact on the performance of the copolymer product, which is reflected in its relative molecular weight, the filtrate loss reduction performance, and the rheological properties of the drilling fluid system. Through comparative experiments, the effect of the monomer ratio on the filtrate loss reduction performance of the product was studied, and the optimal ratio of the monomers in the quaternary copolymer was determined. By keeping the other reaction conditions constant and varying the ratio of acrylamide (AM) to acrylic acid (AA), the filtrate loss performance of the product under different monomer ratios was measured. The FL (API) of the base slurry system after adding the copolymer product is shown in Figure 6. From Figure 6, it can be observed that, as the proportion of AA increases, the FL (API) of the base slurry system gradually decreases when using polymer microsphere filtrate reducers. This is because the number of adsorption and hydration groups in the molecular structure gradually increases, enhancing the filtrate loss reduction performance of the polymer. When the molar ratio of acrylamide (AM) to 2-acrylamido-2-methylpropane sulfonic acid (AMPS) is 10:2.5, the FL (API) of the freshwater base slurry reaches its minimum value of 10.4 mL.

**Figure 7:**
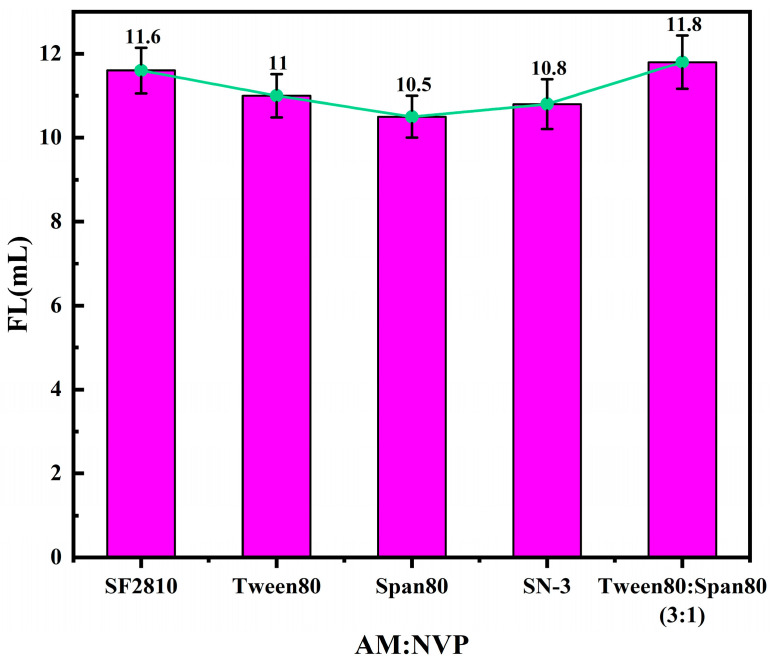
Effects of the AM-to-NVP molar ratio on the performance of fluid loss additives.

By keeping the other reaction conditions constant and varying the ratio of acrylamide (AM) to N-vinylpyrrolidone (NVP), the filtrate loss performance of the quaternary copolymer product under different monomer ratios was measured. The FL (API) of the base slurry system after adding the copolymer product is shown in Figure 7.

From Figure 7, it can be observed that the filtrate loss of the system decreases with the increase in the NVP dosage. When the molar ratio of acrylamide (AM) to N-vinylpyrrolidone (NVP) is 10:2.5, the FL (API) of the base slurry reaches its lowest value of 10.5 mL. However, with further increases in the NVP dosage, the filtrate loss begins to rise. This is because the primary role of NVP is to enhance the rigidity of the polymer’s molecular chain. An appropriate amount of NVP imparts higher chain rigidity to the macromolecule, improving the shear resistance of the copolymer. This prevents the product from degrading during the mixing process, thereby reducing the filtrate loss of the system. However, NVP itself has relatively poor hydration and adsorption properties. When its dosage becomes too high, the number of critical hydration and adsorption groups in the product decreases, reducing its ability to stabilize the colloid. Additionally, the spatial steric hindrance effect caused by the rigid heterocyclic structure of NVP leads to lower reactivity during the reaction. An excessive NVP dosage reduces the overall activity of the reaction system, resulting in a decrease in the molecular weight of the product and a reduction in the number of clay particles that can be adsorbed on a single polymer chain. Consequently, the viscosity of the filtrate decreases, and the filtrate loss of the system increases.

**Figure 8:**
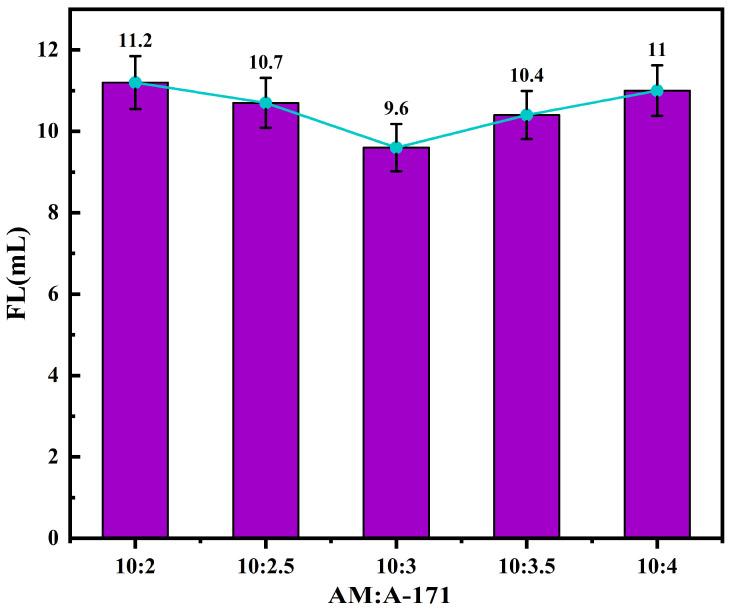
Effects of the AM-to-A-171 molar ratio on the performance of fluid loss additives.

By keeping the other reaction conditions constant and varying the ratio of acrylamide (AM) to A-171, the filtrate loss performance of the quaternary copolymer product under different monomer ratios was measured. The FL (API) of the base slurry system after adding the copolymer product is shown in Figure 8. From Figure 8, it can be observed that as the amount of A-171 increases, the FL (API) of the drilling fluid system initially decreases and then increases. This is because the silanol groups in A-171 undergo chemical condensation with the surfaces of the clay particles, increasing the adsorption rate of the filtrate reducer on the clay particles and thereby reducing the filtrate loss of the base slurry. With a further increase in the A-171 dosage, its encapsulation effect on the clay particles is enhanced, leading to the flocculation of the clay particles, which in turn increases the filtrate loss. When the molar ratio of AM to A-171 is 10:1.5, the filtrate reducer minimizes the FL (API) of the base slurry system to 9.6 mL.

#### 3.1.7. Reaction Time and the Performance of Fluid Loss Additives

**Figure 9:**
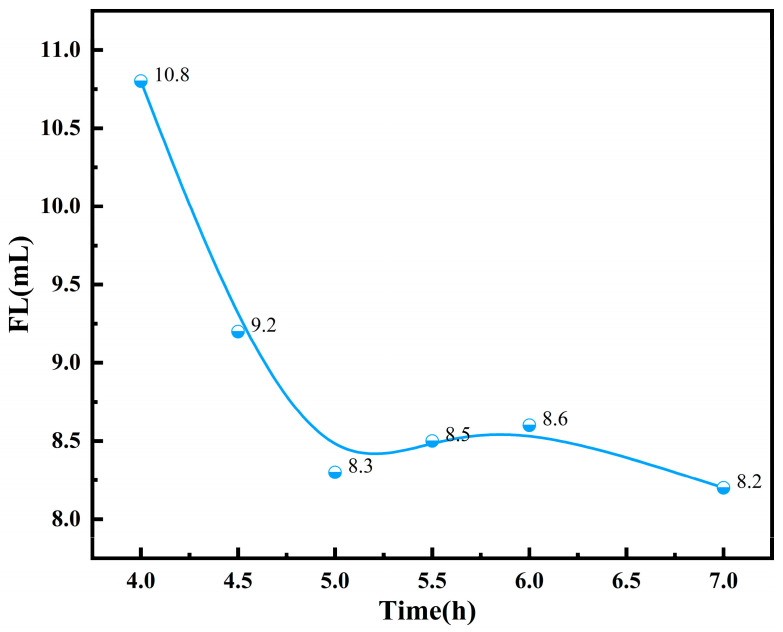
Effects of reaction time on the performance of fluid loss additives.

Since free radical polymerization is an instantaneous, high-speed reaction, the degree of polymerization of the product can reach thousands or even tens of thousands in a very short time, with no intermediate oligomers. Therefore, the reaction time primarily influences the monomer conversion rate, which in turn affects its filtrate loss reduction performance. If the reaction time is too short, the monomer conversion rate will be low, leading to a low product yield. Conversely, if the reaction time is too long, the monomer conversion rate will be nearly complete, and excessively extending the reaction time will only increase the costs. For free radical polymerization, extending the reaction time mainly aims to improve the monomer conversion rate, while it has little effect on the intrinsic viscosity of the polymer. The effect of the polymerization reaction time (t) on the performance of the filtrate reducer was evaluated. By keeping the other reaction conditions constant and varying the polymerization reaction time (t), the FL (API) of the base slurry system after adding the copolymer product was determined, as shown in Figure 9.

From Figure 9, it can be observed that, as the reaction time increases, the filtrate loss of the base slurry system gradually decreases with the addition of the microsphere filtrate reducer. When the reaction time reaches 5 h, the filtrate loss of the polymer product stabilizes, indicating that the copolymer molecules have fully polymerized. At this point, the FL (API) of the base slurry system is at its minimum value of 8.3 mL.

#### 3.1.8. Reaction Temperature and the Performance of Fluid Loss Additives

**Figure 10:**
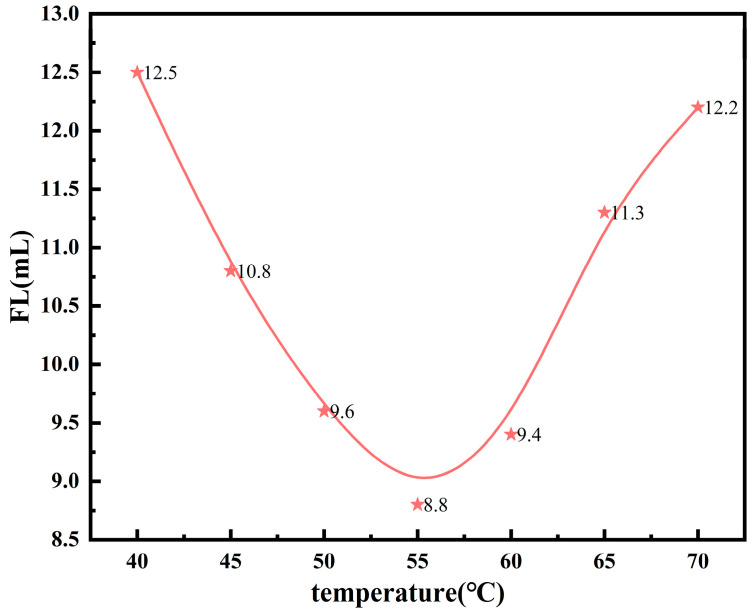
Effects of reaction temperature on the performance of fluid loss additives.

By keeping the other reaction conditions constant and varying the reaction temperature, the effect of the temperature on the performance of the microsphere filtrate reducer was evaluated. The FL (API) of the base slurry system after adding the copolymer product is shown in Figure 10.

From Figure 10, it can be observed that as the reaction temperature increases, the FL (API) of the system initially decreases and then increases, reaching its minimum value at 55 °C. This is because the reaction temperature affects both the polymerization reaction rate and the average degree of polymerization of the product. As mentioned earlier, the polymerization reaction rate increases with the temperature, leading to a higher monomer conversion rate and an increase in the concentration of copolymers in the system, which reduces the filtrate loss. However, the reaction temperature should not be excessively high. When the temperature exceeds 60 °C, the decomposition rate of the initiator becomes too fast, resulting in an excessively high concentration of free radicals in the system. This leads to a decrease in the average degree of polymerization of the copolymer and a corresponding reduction in molecular weight, which is unfavorable for the control of the filtrate loss of the system. Therefore, the optimal reaction temperature is determined to be 55 °C.

The performance of the spherical polymer filtrate reducer is closely related to its synthesis conditions. It is prepared by the reverse microemulsion polymerization method. The effects of various factors on the performance of the filtrate reducer, including the oil-to-water ratio of the microemulsion, the pH value of the reaction system, the amount of emulsifier, the amount of initiator, the total monomer concentration, the monomer ratio, the reaction temperature, and the reaction time, were studied using the single-factor variable method. A freshwater base slurry with bentonite content was used as the testing medium. Based on these experiments, the optimal synthesis conditions were determined, and the FL (API) of the base slurry system after adding the copolymer product was reduced to just 8.2 mL.

### 3.2. Infrared Spectroscopy Analysis

**Figure 11:**
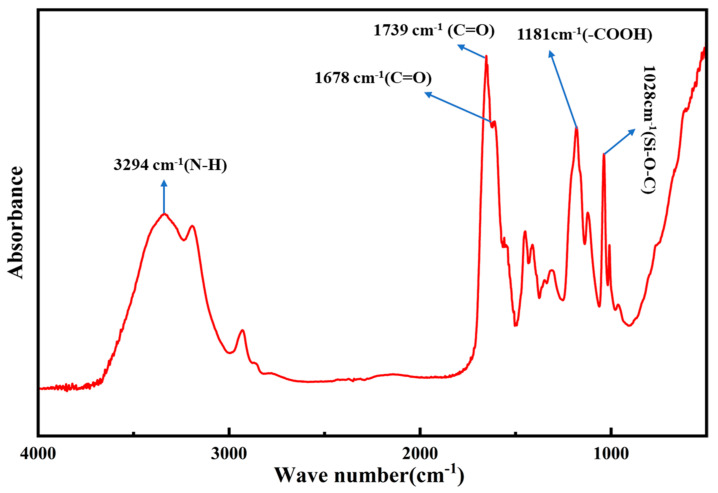
Infrared spectrum of EnSipoly-FL fluid loss additive.

The Nicolet 6700 intelligent Fourier transform infrared spectrometer was used to record the infrared absorption spectrum of the molecule. This was based on the energy level transitions induced by molecular vibrations after the substance absorbed radiant energy. For the analysis, 1–2 mg of the polymer sample was ground into a fine powder in an agate mortar and uniformly mixed with 100–150 mg of dried potassium bromide. The mixture was then placed into a mold and pressed into a pellet using a tablet press for testing. The infrared spectrum was obtained by scanning the sample in the wavelength range of 500 cm^−1^ to 4000 cm^−1^ using the infrared spectrometer. The resulting spectrum is shown in Figure 11.

The filtrate reducer was analyzed using infrared spectroscopy. From Figure 11, the following characteristic absorption peaks can be observed: at 1739 cm^−1^, a C=O absorption peak appears, corresponding to the tertiary amide group (lactam group on the heterocyclic structure); at 1678 cm^−1^, a C=O absorption peak is observed, corresponding to the primary and secondary amide groups; at 3294 cm^−1^, an N-H absorption peak is observed, corresponding to the primary and secondary amide groups; at 1028 cm^−1^, a Si-O-C absorption peak is observed; at 1181 cm^−1^, a carboxyl group absorption peak is observed. These absorption peaks confirm the successful copolymerization of the four monomers. In conclusion, the synthesized product matches the target compound.

### 3.3. Thermal Stability Analysis

**Figure 12:**
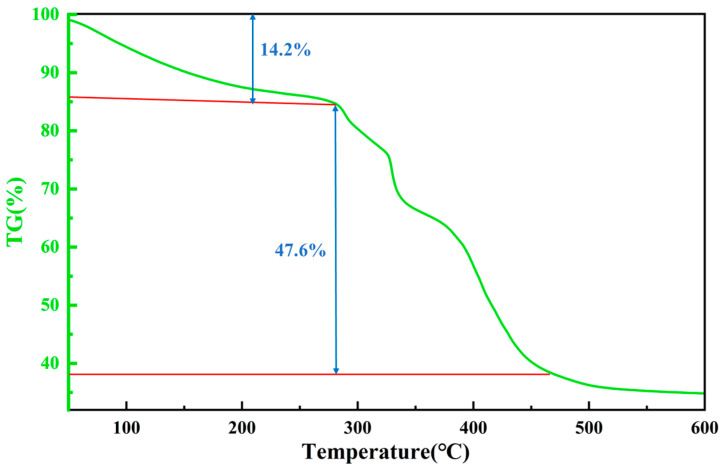
Thermal stability analysis of EnSipoly-FL fluid loss additive.

The thermal stability of the filtrate reducer was analyzed using the HCT-1 thermogravimetric analyzer. The polymer filtrate reducer was placed in the thermogravimetric analyzer, and a thermogravimetric analysis (TGA) was performed under a nitrogen atmosphere. The temperature range for the analysis was set from 20 °C to 600 °C, with a heating rate of 5 °C/min. The TG curve is shown in Figure 12.

From Figure 12, it can be observed that, when the polymer filtrate reducer is heated to 150 °C, its mass loss is minimal. This is primarily due to the strong polar hydrophilic groups in the polymer’s molecular structure, which result in a significant amount of adsorbed and bound water on the molecular surface. During the heating process, this water evaporates first. When the polymer filtrate reducer is heated to 290 °C, its mass loss is only 14.2%. In the temperature range of 150–290 °C, the thermogravimetric curve changes slowly. This is attributed to the evaporation of free water adsorbed by the polar hydrophilic groups, leading to minimal mass loss without any significant breakage of functional groups or side chains. This indicates that the molecule exhibits strong thermal stability. However, in the temperature range of 290–460 °C, the mass loss becomes significant, which is caused by the breakage of side chains and the decomposition of amide and carboxyl groups. Below 290 °C, the polymer filtrate reducer remains stable, demonstrating excellent high-temperature resistance.

### 3.4. Particle Size Analysis

**Figure 13:**
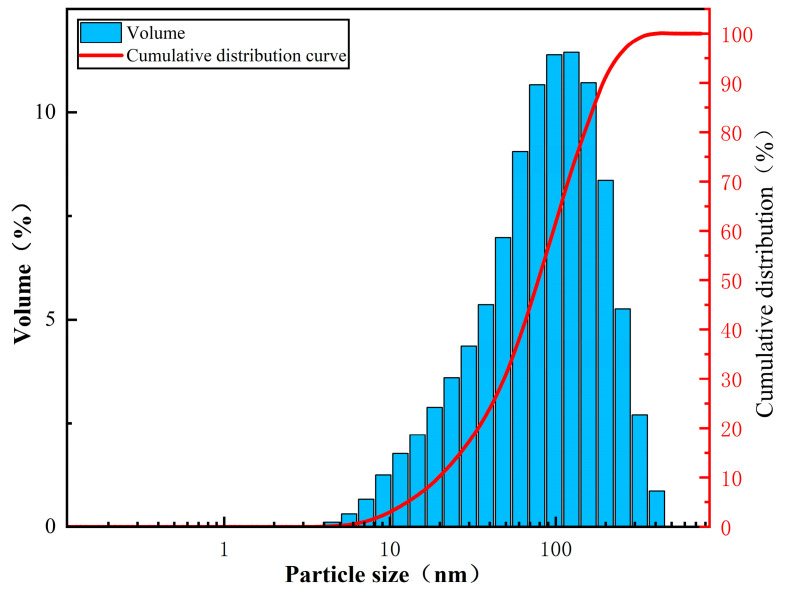
Particle size distribution of EnSipoly-FL fluid loss additive.

The particle size and particle size distribution of the polymer filtrate reducer were measured using a Mastersizer 2000 laser particle size analyzer (manufactured by Zhuhai OMEC Instruments Co., Ltd., China). The results are shown in Figure 13.

From Figure 13, it can be observed that the particle size distribution of the filtrate reducer is relatively narrow (80–300 nm), with an average particle size of 150 nm. The particle size distribution is concentrated, with over 80% of the peak area corresponding to particle sizes in the range of 80–200 nm, indicating that particles of this size constitute the main components of the polymer dispersion system. Additionally, approximately 100% of the particles in the polymer have a particle size of around 1 μm. These micron-sized polymer particles are likely the result of particle aggregation in the solution. Due to the use of reverse microemulsion polymerization technology, the synthesized three-dimensional crosslinked polymer particles exhibit a unique core–shell structure. The outer layer is encapsulated by a large number of hydrophobic chains, and the overlapping and interactions of chains and functional groups may contribute to the increased size of the polymer particles.

### 3.5. Analysis of Molecular Weight of Fluid Loss Additives

The molecular weight of the filtrate reducer EnSipoly-FL was measured using gel permeation chromatography (GPC). The specific experimental steps were as follows. The mobile phase (THF) was degassed under reduced pressure using ultrasonic treatment. Solutions of 5 mL each of polystyrene standards and the sample to be tested were prepared. After characterizing and collecting data using a liquid chromatograph, the GPC test results showed that the number-average molecular weight (Mn) of the filtrate reducer EnSipoly-FL was 5.15 × 10^5^ g/mol.

### 3.6. Fluid Loss Reduction Performance of Fluid Loss Additives

Different concentrations of the filtrate reducer EnSipoly-FL were added to the prepared freshwater base slurry. The rheological properties (at 50 °C) and filtrate loss performance of the drilling fluid were measured before and after aging at 200 °C using a medium-pressure filtration instrument, a rotational viscometer, and a high-temperature and high-pressure filtrate loss apparatus. The results are shown in Table 2.

From Table 2, it can be seen that as the dosage of the filtrate reducer EnSipoly-FL increases, the viscosity and shear strength of the drilling fluid gradually increase, while the filtrate loss decreases. Furthermore, before and after thermal rolling, the changes in the viscosity and shear strength of the drilling fluid are minimal. This indicates that the microsphere filtrate reducer can form a stable network structure with clay particles under high-temperature conditions. Additionally, the filtrate reducer undergoes chemical adsorption with the clay surface through condensation reactions, without significant molecular breakage, thereby reducing the filtrate loss of the drilling fluid. This demonstrates its excellent high-temperature resistance and filtrate loss reduction performance. The primary reason for this performance lies in the molecular structure of the filtrate reducer, which contains numerous heterocyclic groups. These groups enhance the molecular rigidity and increase the spatial steric hindrance, thereby increasing the resistance to thermal motion and conferring high-temperature resistance. The minimal changes in the viscosity and shear strength of the drilling fluid before and after thermal rolling further indicate that the network structure formed between the filtrate reducer and clay particles is stable. The silicon–oxygen groups in the filtrate reducer molecules undergo condensation reactions with hydroxyl groups on the clay surface to form Si-O-Si bonds, which have high bond energies and are resistant to desorption at high temperatures. Additionally, the adsorptive functional groups on the microsphere surface interact with clay particles, improving their dispersibility. The small size and spherical shape of the microsphere particles, combined with their water absorption properties, allow them to swell after absorbing water. This swelling effectively blocks the pores in the mud cake and contributes to forming the mud cake, thereby reducing its permeability.

The high-temperature filtrate reducers Dristemp, DTEMP, and SPNH-HT, both domestic and international, were compared by adding them to the prepared base slurry. The rheological properties (at 50 °C) and filtrate loss performance of the drilling fluid were measured before and after aging at 200 °C using an SD medium-pressure filtration instrument, a ZNN-D6 rotational viscometer, and a GGS42-2 high-temperature and high-pressure filtrate loss apparatus. The results are shown in Table 3.

From Table 3, it can be observed that, when evaluating the filtrate loss performance of different filtrate reducers at the same dosage in the base slurry, the rheological parameters of the drilling fluid are similar before thermal rolling. However, after thermal rolling, significant differences are observed in the rheological performance of the drilling fluids. The viscosity and shear strength of the drilling fluids prepared with the three comparison filtrate reducers decrease significantly after thermal rolling. This indicates that the network structure formed between these filtrate reducers and the clay particles is destroyed, leading to the aggregation of clay particles under high temperatures. Additionally, the filtrate loss of the drilling fluids prepared with SPNH-HT and DTEMP is relatively high. This may be due to the desorption of the filtrate reducer from the clay particles and the thermal degradation or molecular breakdown of the filtrate reducer under high temperatures. In comparison to the three filtrate reducers, EnSipoly-FL demonstrates superior high-temperature resistance and filtrate loss reduction performance.

### 3.7. Evaluation of Biodegradability of Fluid Loss Additives

Biodegradability Test: The test was conducted by referring to national standard documents such as the Determination of Five-Day Biochemical Oxygen Demand (BOD5) in Water—Dilution and Seeding Method (GB7488-87 [54]). In this test, the ratio of the measured BOD5 data to the detected COD is used as the basis for the analysis of the biodegradability of the filtrate reducer. When the BOD5/COD ratio is less than 0.05, the material is considered difficult to degrade. When the ratio is greater than 0.1, the material is considered degradable. When the ratio is greater than 0.25, the material is considered easily degradable. The higher the BOD5/COD ratio, the more easily the material degrades, indicating better biodegradability.

From Table 4, it can be seen that, upon testing the biochemical oxygen demand (BOD5) of EnSipoly-FL solutions at concentrations of 0.1%, 0.5%, 1.0%, and 2.0%, the BOD5/COD ratio is approximately 0.2 across different concentrations. This indicates that EnSipoly-FL is biodegradable and exhibits strong environmental acceptability.

### 3.8. Evaluation of Biotoxicity of Fluid Loss Additives

To conduct the toxicity test, laboratory-grown brine shrimp were used, or commercial brine shrimp eggs were hatched. The eggs were added to diluted water at a concentration of 1.0 g/L and incubated under conditions of 25 °C, salinity of 30–35, and a light intensity of 2000–3000 lx with aeration. Nauplii hatched from the same batch were used for the toxicity experiment. The survival counts of brine shrimp in the EnSipoly-FL group are shown in Table 5.

**Figure 14:**
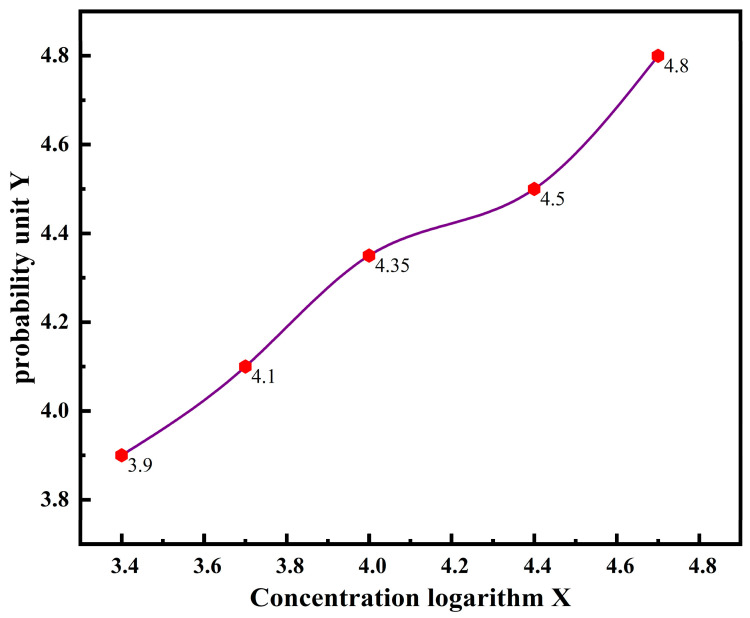
Relationship between logarithmic concentration and mortality probability in Group A.

To analyze the data from Group A, the concentration logarithm was used as the *X*-axis, and the corrected mortality probability unit was used as the *Y*-axis. The results are shown in Figure 14.

If the probability unit Y = 5, the value of X is lg LC_50_, and the LC_50_ value of EnSipoly-FL is 64,605.6 mg/L. Experimental and data analysis: The LC_50_ value of the biotoxicity of the fluid loss reducer was 64,605.6 mg/L, much higher than 3000 mg/L, meeting the requirements of environmental protection.

## 4. Conclusions

(1) Through a carefully designed reverse microemulsion polymerization strategy, a novel, environmentally friendly polymer filtrate reducer was successfully developed. This reducer demonstrated exceptional filtrate loss control capabilities in high-temperature drilling fluids while meeting environmental protection standards.

(2) A systematic study determined the optimal synthesis parameters, including an initiator dosage of 0.4%, a monomer molar ratio of AM:AA:NVP:A-171 = 10:2.5, suitable reaction conditions of 60 °C for 5 h, 56 mL of heptane as the oil phase, 20 g of emulsifier (Tween 80/Span 80 = 3:1), a stable pH value of 7, and 45 mL of deionized water as the aqueous phase, ensuring the optimal performance of the product.

(3) Infrared spectroscopy, nuclear magnetic resonance spectroscopy, and thermogravimetric analysis indicated that the synthesized product possessed the expected core–shell structure, strong molecular chain rigidity, excellent thermal stability, and a uniform particle size distribution. These characteristics result in good heat resistance, ensuring high-efficiency fluid loss performance in drilling fluids.

(4) This environmentally friendly polymer fluid loss additive has shown great potential in drilling fluid applications. Particularly under high-temperature and harsh geological conditions, its outstanding performance suggests promising prospects for widespread use in drilling operations.

## Figures and Tables

**Table 1:** Effects of different oil-to-water ratios on the stability of the inverse microemulsion system.

Number	Oil–Water Ratio	Phenomenon
1	1:4	turbid
2	2:4	no obvious phase separation
3	3:4	no obvious phase separation
4	4:4	layering
5	5:4	uniform and transparent

**Table 2:** Effects of fluid loss additive EnSipoly-FL on the properties of the base slurry.

Dosage of Fluid Loss Reducer	State	AV/ (mPa·s)	PV/ (mPa·s)	YP/ (Pa)	FL_API_ /mL	FL_(HTHP)_ /mL
0	Before thermal aging test	13	10	3		
	After thermal aging test	9	7	2	63.7	-
1%	Before thermal aging test	17	15	2		
	After thermal aging test	16	14	2	13.8	51.2
2%	Before thermal aging test	23	18	5		
	After thermal aging test	21	18	3	8.4	32.8
3%	Before thermal aging test	26	20	6		
	Before thermal aging test	23	19	4	6.3	17.6

Note: FL(HTHP) is measured at 175 °C.

**Table 3:** Effects of fluid loss additives on the properties of the base slurry.

Fluid Loss Reducer	State	AV/ (mPa·s)	PV/ (mPa·s)	YP/ (Pa)	FLAPI /mL	FL(HTHP) /mL
SPNH-HT	Before thermal aging test	20	16	4		
	After thermal aging test	8	6	2	62.8	-
Dristemp	Before thermal aging test	23	17	6		
	After thermal aging test	14	11	3	11.3	48.2
DTEMP	Before thermal aging test	24	21	3		
	After thermal aging test	11	9	2	31.6	-
EnSipoly-FL	Before thermal aging test	24	20	4		
	Before thermal aging test	25	22	3	7.3	18.8

**Table 4:** Biodegradability evaluation of EnSipoly-FL.

Concentration (%)	COD (mg/L)	BOD5 (mg/L)	BOD5/COD
0.1	23	5.1	0.221
0.5	126	26.9	0.213
1.0	281	61.1	0.217
2.0	608	128.6	0.211

**Table 5:** Biotoxicity of fluid loss additives.

Group	Concentration (mg/L)	Logarithm of Concentration (X)	Number of Experimental Organisms (ind)	Number of Deaths (r)	Mortality Rate (%)	Corrected Mortality Rate (%)	Probability Unit (Y)
A1	/	/	40	6	15	/	/
A2	2500	3.3979	40	10	25	11.8	3.8150
A3	5000	3.6990	40	12	30	17.6	4.0693
A4	10,000	4	40	14	35	23.5	4.2775
A5	20,000	4.3010	40	17	42.5	32.4	4.5435
A6	40,000	4.6021	40	20	50	41.2	4.7776

## Data Availability

The original contributions presented in this study are included in the article. Further inquiries can be directed to the corresponding author.
